# In Vitro Antibacterial and Antiproliferative Potential of *Echinops lanceolatus* Mattf. (Asteraceae) and Identification of Potential Bioactive Compounds

**DOI:** 10.3390/ph13040059

**Published:** 2020-03-30

**Authors:** Armel Jackson Seukep, Yong-Li Zhang, Yong-Bing Xu, Ming-Quan Guo

**Affiliations:** 1 CAS Key Laboratory of Plant Germplasm Enhancement and Specialty Agriculture, Wuhan Botanical Garden, Chinese Academy of Sciences, Wuhan 430074, China; seukepp@yahoo.fr (A.J.S.); zhangyongli@wbgcas.cn (Y.-L.Z.); xuyongbing17@mails.ucas.ac.cn (Y.-B.X.); 2 Department of Biomedical Sciences, Faculty of Health Sciences, University of Buea, P.O. Box 63, Buea, Cameroon; 3 Sino-Africa Joint Research Center, Chinese Academy of Sciences, Wuhan 430074, China; 4 Innovation Academy for Drug Discovery and Development, Chinese Academy of Sciences, Shanghai 201203, China

**Keywords:** *Echinops lanceolatus*, antimicrobial, cytotoxicity, UPLC–ESI–MS/MS

## Abstract

Many species belonging to the genus *Echinops* are widely used in traditional medicine to treat infectious diseases and cancers. The present study aimed to evaluate the antibacterial and antiproliferative properties of *Echinops lanceolatus* Mattf. (Asteraceae). The activity of the methanolic extract and subsequent partition fractions was investigated against drug-resistant bacteria (Gram-negative and Gram-positive) and human tumor cell lines using broth microdilution and sulforhodamine B (SRB) assay, respectively. Our findings revealed weak to moderate antibacterial activities of tested extracts, with the recorded minimal inhibitory concentrations ranging from 256 to 1024 µg/mL. The ethyl acetate fraction (EL-EA) was found to be the most effective. Likewise, that fraction displayed strong antiproliferative potential with recorded IC_50_ of 8.27 µg/mL and 28.27 µg/mL on A549 and HeLa cells, respectively. An analysis based on the ultra-performance liquid chromatography–electrospray ionization tandem mass spectrometry (UPLC–ESI–MS/MS) of the EL-EA fraction allowed the identification of 32 compounds, of which quinic acid and derivatives, cinnamic acid derivatives, dihydrokaempferol, naringenin-7-*O*-glucoside, apigenin-7-*O*-d-glucoside, naringin, apigenin, rhoifolin, coniferyl aldehyde, and secoisolariciresinol are well-known compounds of biological importance. This study is first to report on the biological activity and phytochemical profile of *E. lanceolatus*. We provide a baseline to consider *E. lanceolatus* as a valuable source of anti-infective and antiproliferative agents.

## 1. Introduction

The resurgence of drug-resistant infectious diseases, as well as cancers, propels the scientific community to seek alternative medicine. This double burden is indeed a serious public health-threatening worldwide [1]. As part of the implementation of the Global Action Plan on antimicrobial resistance (AMR), the World Health Organization (WHO) caught up with a list of priority antibiotic-resistant pathogens to guide research into and the discovery and development of new therapeutic agents. Some bacteria, listed as a critical priority, are the members of the group named ESKAPEE (*Enterococcus* spp., *Staphylococcus aureus*, *Klebsiella pneumoniae*, *Acinetobacter baumannii*, *Pseudomonas aeruginosa*, *Enterobacter* spp., and *Escherichia coli*) [2,3]. Yet, alternative strains like *Salmonella* species are also of clinical concern. Botanicals are broadly recognized as some of the most prolific sources of leads for the development of new drug candidates. They cover a large spectrum of therapeutic uses with a great diversity of chemical structures. The scientific community still considers exploring medicinal plants for new drug discovery. The application of molecular, analytical, and computational techniques has raised the availability of novel compounds that can be handily isolated from natural sources [4,5]. Many species memberships to the genus *Echinops* are used in traditional medicine, primarily in Africa and Asia, to treat various ailments including infectious and respiratory diseases, inflammation, and to relieve pain. They are also claimed to have aphrodisiac properties, to fasten expulsion of the placenta, and to act on the kidney for the elimination of renal stones. More than 151 compounds have been isolated from the *Echinops* genus including thiophenes, terpenes, flavonoids, and other phenolic compounds, phenylpropanoids, alkaloids, and lipids [6]. Varied extracts, isolated compounds, and essential oils from members of this genus were found to exert multiple biological properties including anti-infective, antiproliferative, antioxidant, and anti-inflammatory [6]. However, several species belonging to this genus are claimed to have traditional medicinal uses but their biological effect not yet been investigated. It is the case of the member examined in the present study namely *Echinops lanceolatus*.

*Echinops lanceolatus* Mattf. (Asteraceae) is a native plant to Cameroon, Central African Republic, and Nigeria [7]. *E. lanceolatus* is a spiny perennial herb, chamaephytes, with marcescent leaves. It is a much-branched herb with stout stems 2–3 ft. high from a woody stock. Florets are blue or white in spherical heads up to 2 in. across. This plant species is widely distributed in open grassland including West Tropical Africa [7,8]. To the best of our knowledge, no biological activities, nor phytochemical studies have been reported on this plant species. In the present investigation, as part of our continuing search for potent therapeutic agents from natural sources, we assessed their antibacterial and antiproliferative potential. Assays were performed against the ESKAPEE group’s bacteria and *Salmonella enterica*, and human tumor cell lines (HepG2, HeLa, HT-29, and A549). The ultra-performance liquid chromatography (UPLC) coupled to electrospray ionization tandem mass spectrometry (ESI–MS/MS) analysis was applied for the identification of the potential bioactive chemical components of the ethyl acetate fraction (EL-EA), the most active extract. Compounds were tentatively identified based on the comparison of their ESI-MS^2^ data with the corresponding standards and fragmentation pathways from databases (ChemSpider, HMDB, PubChem, mzCloud, and mzVault) and/or data available in the literature. To the best of our knowledge, the present study reports for the first time the biological properties of *E. lanceolatus* and their phytochemical profile. This, therefore, provides a baseline for thorough investigations for the isolation, purification, and development of potent phytomedicine against the double burden of infectious diseases and tumors.

## 2. Results

### 2.1. Antibacterial Potential

Results of bacterial susceptibility testing to *E. lanceolatus* extracts are summarized in Table 1. Methanolic extract (EL-MeOH) and fractions depicted selective inhibitory effects depending on the studied bacteria strain, with minimal inhibitory concentrations (MICs) varying from 256 to 1024 µg/mL. The lowest MIC value of 256 µg/mL was recorded with EL-EA against *S. enterica*. EL-EA exhibited the highest spectrum of inhibition, acting against 6/8 of studied bacteria, followed by the dichloromethane (EL-DCM) fraction (5/8). EL-MeOH and other fractions (*n*-hexane and *n*-butanol) exerted the activity on 4/8 bacteria strains. Interestingly, EL-MeOH, and all subsequent partition fractions, prevented the growth of *S. enterica*, *E. cloacae*, and *S. aureus*. A bactericidal effect (minimal bactericidal concentration (MBC) = 1024 µg/mL) was recorded with EL-EA on *S. aureus*.

### 2.2. The Antiproliferative Potential of E. lanceolatus

**Figure 1:**
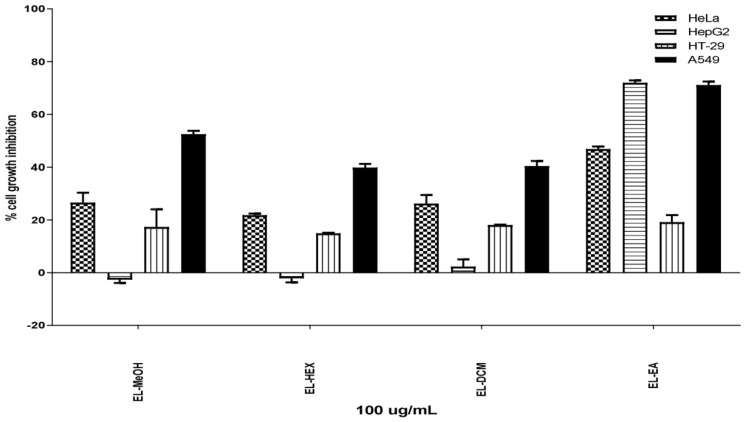
Percentage of cell growth inhibition of *E. lanceolatus* extracts. Data are expressed as mean ± SEM, *p* < 0.05.

The percentage of tumor cell growth inhibition of studied samples is shown in Figure 1. At a fixed concentration of 100 µg/mL, EL-MeOH and fractions from *E. lanceolatus* displayed antiproliferative activity depending on studied cells. EL-EA was found to be the most potent, displaying significant inhibition of 72% and 71% on HepG2 and A549, respectively.

**Figure 2:**
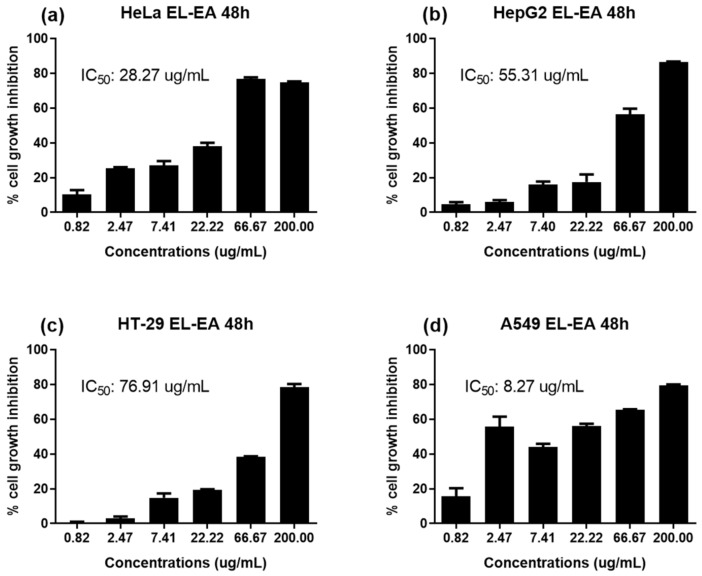
Concentration-dependent antiproliferative activities of EL-EA against (**a**) HeLa, (**b**) HepG2, (**c**) HT-29, and (**d**) A549 human tumor cell lines. Cells incubated with extract for 48 h. IC_50_: half-maximal inhibitory concentration. EL-EA: *E. lanceolatus* ethyl acetate fraction. HepG2 (human liver cancer cell line), HeLa (cervical cancer cells), HT-29 (human colon cancer cell line), and A549 (adenocarcinomic human alveolar basal epithelial cells). Data are expressed as mean ± SEM, *p* < 0.05.

Furthermore, a concentration-dependent effect of EL-EA (tested at concentrations ranging from 0.82 to 200 µg/mL) was studied on HepG2, A549, HeLa, and HT-29 cell lines for 48 h. The results revealed a dose-dependent inhibition of tumor cell growth (Figure 2). The IC_50_ values ranged from 8.27 to 76.91 µg/mL. The lowest IC_50_ value (significant activity) of 8.27 µg/mL recorded on A549.

### 2.3. UPLC–ESI–MS/MS Analysis of EL-EA Fraction

**Figure 3:**
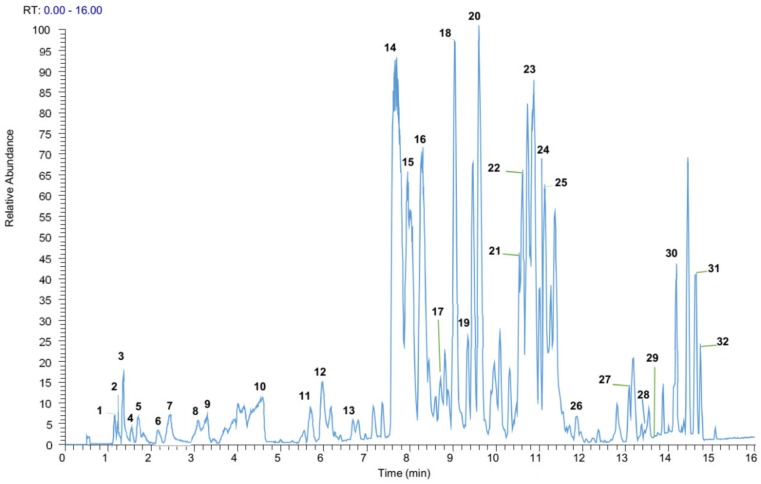
Ultra-performance liquid chromatography–electrospray ionization tandem mass spectrometry (UPLC–ESI–MS/MS) base peak chromatogram of the *E. lanceolatus* EA fraction. The peak numbers in this figure correspond to those used in Table 2.

**Figure 4:**
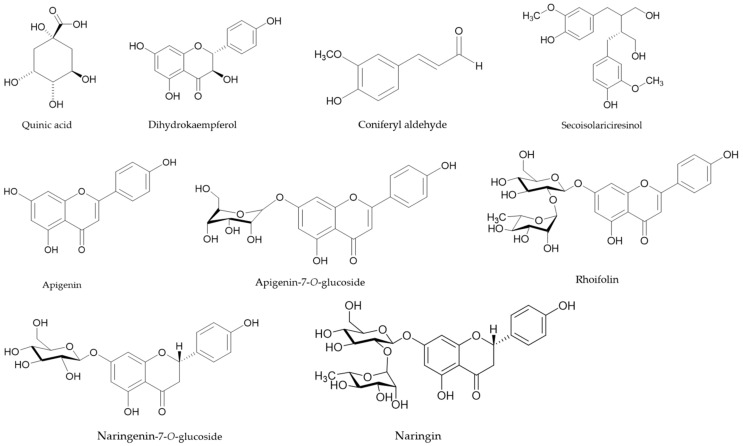
The chemical structures of some well-known identified compounds of biological importance from the *E. lanceolatus* EA fraction.

To explore the potential bioactive compounds in the EL-EA fraction, which may be responsible for the recorded biological activities, a study of the EL-EA fraction was conducted based on UPLC–ESI–MS/MS in the negative ion mode. The analysis led to the detection of several peaks in that fraction, of which 32 compounds were identified with high-resolution MS and MS/MS data (Figure 3). Compounds corresponding to Peaks 1 to 24 were identified by comparison of the mass spectra with the available standards from databases (ChemSpider, mzCloud, mzVault, HDMB, and PubChem) and the literature data; identification of Peaks 25 to 32 was only based on available standards from databases. The retention times (Rt), molecular formula, and MS^2^ data are condensed in Table 2. Some major peaks found between 9 and 10, 13 and 14, 17 and 18, 19 and 20, 20 and 21, 22 and 23, 25 and 26, 26 and 27, 27 and 28, 29 and 30, 30 and 31, and after Peak 31 (Figure 1) were not identified. Subject to further studies, we can hypothesize that the corresponding unidentified compounds may be new. The phytochemicals successfully identified were mainly phenolic acids, polyphenols (flavonoids and lignan), organic acids, and fatty acids, as detailed below. The chemical structures of some well-known identified compounds of biological importance are shown in Figure 4.

#### 2.3.1. Phenolic Acids

Peak 2 was identified as d-(-)-quinic acid, which showed an [M-H]^−^ ion at *m/z* 191.0551. Identified quinic derivatives (or conjugates) included 5-coumaroylquinic acid, which showed an [M-H]^−^ ion at *m/z* 337.0924 (Peak 12), a 5-feruloyl quinic acid [M-H]^−^ ion at *m/z* 367.1027 (Peak 13), a dicaffeoylquinicacid (DCQ) isomer 1 [M-H]^−^ ion at *m/z* 515.1187 (Peak 14), a DCQ isomer 2 [M-H]^−^ ion at *m/z* 515.1186 (Peak 15), and a DCQ isomers 3 and 4 [M-H]^−^ ion at *m/z* 515.1189 (Peaks 16 and 17, respectively). Other phenolic acids included a dihydroxybenzoic acid [M-H]^−^ ion at *m/z* 153.0181 (Peak 3), a hydroxy-methoxy-benzoic acid isomers 1 and 2 [M-H]^−^ ion at *m/z* 167.0338 (Peaks 4 and 6, respectively), and a 3-hydroxybenzoic acid [M-H]^−^ ion at *m/z* 137.0231 (Peak 11). The identified cinnamic acid derivatives comprised dihydroxycinnamic acid isomers 1 (Peak 5, [M-H]^−^ ion at *m/z* 179.0339) and 2 (Peak 7, [M-H]^−^ ion at *m/z* 179.0338), and a coumaric acid isomers 1 and 2 [M-H]^−^ ion at *m/z* 163.0389 (Peaks 8 and 9, respectively). Peak 21 was identified as coniferyl aldehyde, which showed an [M-H]^−^ ion at *m/z* 177.0546.

#### 2.3.2. Polyphenols

##### Flavonoids

Peak 18 was identified as dihydrokaempferol ([M-H]^−^ ion at *m/z* 287.0556), a flavanonol. Peaks 19 and 22 were two flavanone glycosides, namely a naringenin-7-*O*-glucoside (prunin) ([M-H]^−^ ion at *m/z* 433.1134) and a naringin ([M-H]^−^ ion at *m/z* 579.1503), respectively. Peak 24 was identified as an apigenin ([M-H]^−^ ion at *m/z* 269.0451). Two other memberships of apigenin family have been also detected, corresponding to Peak 20 (apigenin-7-*O*-β-d-glucoside (cosmosiin or apigetrin), [M-H]^−^ ion at *m/z* 431.0977) and Peak 23 (rhoifolin, [M-H]^−^ ion at *m/z* 577.1346).

##### Lignan

Peak 26 was identified as (-)-secoisolariciresinol, which showed an [M-H]^−^ ion at *m/z* 361.0818.

#### 2.3.3. Organic Acids and Fatty Acids

The identified organic acids included carboxylic acids namely a succinic acid [M-H]^−^ ion at *m/z* 117.0180 (Peak 1), a nonanedioic acid [M-H]^−^ ion at *m/z* 187.0965 (Peak 10), and a cyclandelate [M-H]^−^ ion at *m/z* 275.1647 (Peak 29). The fatty acids found were a (-)-pinellic acid [M-H]^−^ ion at *m/z* 329.2328 (Peak 25), polyunsaturated fatty acids including a 13-KODE (13-Keto-9Z,11E-octadecadienoic acid) [M-H]^−^ ion at *m/z* 293.21 (Peak 27), a 13-HODE (13S-hydroxyoctadecadienoic acid) [M-H]^−^ ion at *m/z* 293.2117 (Peak 28), and an alpha-linolenic acid [M-H]^−^ ion at *m/z* 277.2166 (Peak 30, tentatively). Peak 31 was tentatively identified as an ethyl myristate [M-H]^−^ ion at *m/z* 255.2324, a long-chain saturated fatty acid. Peak 32 was tentatively identified as an oleic acid [M-H]^−^ ion at *m/z* 281.2479, a C18 monounsaturated fatty acid.

## 3. Discussion

Our study included the ESKAPEE group’s bacteria and *S. enterica*. These bacteria are known to express high resistance to traditional antibiotics [3,22]. The WHO [2], in the context of research for efficient therapeutic agents against AMR, gave priority to these bacteria of clinical concern for antibacterial research. These, therefore, represent the good models in the search of alternative medicine to combat drug resistance. The antibiotic resistance profile of studied bacteria was ascertained during preliminary investigations (data not shown). Medicinal plant extracts are routinely classified as highly active if the MIC < 100 μg/mL, moderately active when 100 ≤ MIC ≤ 625 μg/mL, and weakly active if MIC ≥ 625 µg/mL [23]. According to these interpretive criteria, it can be deduced that EL-MeOH and the subsequent partition fractions displayed weak to moderate antibacterial activities based on MIC values obtained. The moderate activity was recorded with EL-EA against *S. enterica* (MIC = 256 µg/mL), *E. cloacae,* and *S. aureus* (MIC = 512 µg/mL); EL-DCM against *S. aureus* (MIC = 512 µg/mL); EL-Hex and EL-BuOH fractions against *S. enterica* and *E. coli* (MIC = 512 µg/mL), respectively (Table 1). The EL-MeOH, as a complex mixture of chemical components, was less active than derived fractions. These indicate that partitioning by successive depletion enhances the biological activity of *E. lanceolatus*. All fractions were found effective against *S. enterica* and *S. aureus*. These bacteria are common food-borne pathogens causing gastrointestinal (GIT) troubles [24]. The present study underscores evidence of the traditional use of *Echinops* L. in the treatment of GIT troubles. Most of the studied bacterial strains were Gram-negative. The latter is found most resistant compared to their counter-part Gram-positive ones, owed to the presence of an outer membrane. The outer membrane gives Gram-negative bacteria an increased ability to reduce the penetration of antibacterial molecules across the membrane, leading to intrinsic resistance to many molecules including therapeutic agents [25,26]. The differences of sensitivity recorded for the same extract with different bacteria strains could be due to intrinsic differences in the chemical composition of the bacterial cell wall [26]. Otherwise, the differences obtained for the same bacterium and the different fractions suggest the qualitative and quantitative differences in antibacterial active principles or different action mechanisms of biologically active components.

The cytotoxicity of a plant extract on cancer cell lines is considered significant or strong when IC_50_ < 20 µg/mL, moderate if 20 µg/mL < IC_50_ < 50 µg/mL, low if 50 µg/mL < IC_50_ < 200 µg/mL, and no cytotoxicity if IC_50_ > 200 µg/mL [27]. Based on these cut-off points, the EL-EA fraction depicted strong cytotoxicity against adenocarcinomic human alveolar basal epithelial cells A549 (IC_50_ = 8.27 µg/mL). In addition, the same fraction showed moderate cytotoxicity on HeLa (IC_50_ = 28.27 µg/mL). As previously observed with antibacterial activities, EL-EA displayed noteworthy anticancer potential, suggesting their potential use in the fighting against the double burden of infectious diseases and cancers. To the best of our knowledge, the present investigation reports for the first time the antibacterial activity and antiproliferative potential of *E. lanceolatus*. However, some species of the genus *Echinops* (Asteraceae), such as *Echinops giganteus* and *Echinops grijsii*, are well known to have significant anti-tumor and anti-infective effects [6]. The present study provides additional information on the antibacterial and cytotoxic properties of the aerial parts of *E. lanceolatus*, membership of the *Echinops* genus and Asteraceae family. The biological properties of *E. lanceolatus,* and particularly the EL-EA fraction recorded, could be attributed to the presence of structurally diverse secondary metabolites. Several phytochemicals have displayed functional activities that imply they could be responsible for a significant role in preventing a broad range of chronic diseases. However, the bioactivity of a plant extract does not depend exclusively on the presence of secondary metabolites. Indeed, the quantity, the quality, the type of extraction solvent, and possible interactions between the different constituents are some factors, which can also influence the activities [28,29,30,31,32]. The UPLC–ESI–MS/MS analysis of EL-EA revealed the presence of many compounds of which 32 have been identified, as shown in Table 2. The identified chemical compounds were mainly phenolic acids, flavonoids, lignan, organic acids, and fatty acids. The biological effects of these groups of compounds are no longer to be demonstrated. The bioactivity of identified compounds has been widely reported in the literature.

Some of the identified compounds including quinic acid, apigenin, apigenin-7-*O*-glucoside, kaempferol, and dicaffeoylquinic acid are common to members of the *Echinops* genus and Asteraceae family [6]. Several studies documented on biological activities (including antimicrobial and antiproliferative properties) of organic acids (succinic and dihydrobenzoic acids) [33,34,35,36] and phenolic compounds (cinnamic acid derivatives, quinic acid and derivatives) [37,38,39,40,41,42] identified in the present work. The flavonoids identified are not to be outdone. Flavonoids represent the largest group of naturally occurring polyphenols. They are common plant secondary metabolites widely used in phytomedicine to cure a wide range of ailments. Some are well-known antiproliferative phytochemicals such as dihydrokaempferol [43], flavanones glycosides (naringenin-7-*O*-glucoside, apigenin-7-*O*-β-d-glucoside, and naringin) [44,45,46,47,48]. Apigenin is another flavonoid class compound reported to exhibit several biological functions such as antibacterial, antiviral, anti-inflammatory, and antioxidant activities [49,50]. A well-known member of the apigenin family, with substantial biological properties, is the tri-substituted flavone rhoifolin [51], also identified in the present investigation. Other compounds identified including the phenylpropanoid coniferyl aldehyde [52], the dibenzyl butanediol lignan (-)-secoisolariciresinol [53,54], and fatty acids [55] have been revealed to possess valuable biological benefit against microbes and malign cells.

The biological activities of secondary metabolites identified in the present study underscore the correlation of the presence of these phytochemicals in *E. lanceolatus* and their antimicrobial and antiproliferative potential obtained against studied drug-resistant bacteria and human tumor cell lines (HeLa, A549, HepG2, HT-29), respectively. Each constituent could act by interacting with other constituents of the mixture, leading to the recorded activities. However, we can also hypothesize about the individual action of some compounds. The present study gives evidence of *E. lanceolatus* as a plant of pharmaceutical importance, a valuable source of biologically active compounds. Thorough investigations will allow the isolation and purification of each component along with an evaluation of their biological properties and action mechanisms.

## 4. Materials and Methods

### 4.1. Chemicals and Reagents

Trypticase Soy Agar (TSA) and Trypticase Soy Broth (TSB) (Qingdao Hope Bio-Technology, Qingdao, China) were used for bacteria culture. *para*-Iodonitrotetrazolium chloride (INT 98%, Macklin, Shanghai, China) served as a bacterial growth indicator. Dimethyl sulfoxide (DMSO ≥99.0%) obtained from Sinopharm Chemical Reagent (Shanghai, China) was used to dissolve extracts. Streptomycin (purity >98%, Abmole Biosciences, Houston, TX, USA) was used as the positive control for bacterial susceptibility testing. Dulbecco’s Modified Eagle Medium (DMEM, Gibco, Beijing, China), Minimum Essential Medium Eagle (MEM, Hyclone, Logan, UT, USA), McCoy’s 5A (Boster Biological Technology Co., Ltd., Pleasanton, CA, USA), and Ham’s F12 K (Procell Life Science & Technology Co., Ltd., Wuhan, China) were used for cancer cells culture. Other reagents for cell culture including fetal bovine serum (FBS), Sulforhodamine B (SRB), glutamine, and penicillin were obtained from Sigma-Aldrich (St. Louis, MO, USA).

### 4.2. Plant Material

The aerial parts of *Echinops lanceolatus* Matt F. (Asteraceae) were collected in Bangangté (West Region, Cameroon), coordinates (5.1444° N, 10.5240° E), in July 2018. The fresh plant was cleaned with water, cut into fine parts, and then air-dried away from direct sunlight. Next, the air-dried plant was crushed and the resulting powder packed for further experiments. The plant sample was identified and authenticated at the National Herbarium of Cameroon (HNC, Yaoundé, Cameroon) with the kind assistance of M. Eric Ngansop (Taxonomist, HNC), where a voucher specimen was lodged under a reference number (14148SRF-CAM and 35113/HNC).

### 4.3. Extraction Procedure

Air-dried powder (100 g) of plant sample was macerated into methanol (MeOH) for 24 h, followed by ultrasound-assisted extraction (KQ-500DE, Kunshan Ultrasonic Instrument Co., Ltd.) for 30 min. The mixture was filtered using Whatman paper grade 1. The same procedure was repeated twice with the remaining residue. The overall filtrate was evaporated under vacuum in a rotary evaporator at reducing pressure and temperature (below 45 °C) to yield 7.5 g of an oily dark extract. The partitioning was carried out according to the scheme previously described [56]. In brief, MeOH extract was suspended in deionized water to make a 95% aqueous solution. Afterward, a successive depletion was performed using solvents of increasing polarity including *n*-hexane, dichloromethane (DCM), ethyl acetate (EA), *n*-butanol (*n*-BuOH), and finally water (H_2_O), to afford fractions. Corresponding fractions were evaporated *in vacuo* to yield the residues of 0.84 g, 0.56 g, 0.35 g, 1.85 g, and 2.3 g, respectively. MeOH extract and fractions were kept at 4 °C for future uses.

### 4.4. UPLC–ESI–MS/MS Analysis

A Q Exactive Orbitrap^®^ LC-MS/MS (Thermo Fisher Scientific, San Jose, CA, USA) mass spectrometer coupled with a Vanquish UPLC system (Thermo Fisher Scientific, San Jose, CA, USA) was employed for the LC-MS analysis, which was equipped with an ESI source. The separation of the EL-EA fraction was acquired with a Hypersil Gold column (C18) column (150 × 2.1 mm, 5 μm) at 25 °C. The injection volume of the sample was 1 µL. Scan range *m/z* 70-1050; ESI Spray Voltage: 3.2 kV; Sheath gas flow rate: 35 (arbitrary units); Aux Gas flow rate: 10 (arbitrary units); Capillary Temp: 320 °C. Polarity: negative; MS/MS data-dependent scans. The mobile phase B and the used gradient condition are shown in Table 3.

Compounds in EL-EA were identified by comparing the retention time, parent ion, and mass fragments with available standards in databases (ChemSpider, mzCloud, mzVault, HMDB, and PubChem) and/or literature data [9,10,11,12,13,14,15,16,17,18,19,20,21].

### 4.5. Antibacterial Assay

#### 4.5.1. Bacterial Strains

Eight drug-resistant bacterial strains were examined for their sensitivity to crude MeOH extract and fractions. They were obtained from CCTCC (China Center for Type Culture Collection), CMCC (Center for Medical Culture Collection), and ATCC (American Type Culture Collection). These strains included two Gram-positive (*Enterococcus faecalis* ATCC29212 and *Staphylococcus aureus* CCTCC AB91093) and six Gram-negative bacteria (*Acinetobacter baumannii* ATCC19606, *Enterobacter cloacae* ATCC700323, *Escherichia coli* CCTCC AB93154, *Klebsiella pneumoniae* CMCC(B)46117, *Pseudomonas aeruginosa* ATCC9027, and *Salmonella enterica* CCTCC AB94018). The WHO [2] has classified the selected strains as priorities in the research for antibacterial agents.

The studied bacteria were maintained on agar slant at 4 °C and each bacteria strain was subcultured (activation) at 37 °C for 18–24 h on fresh appropriate agar plates 24 h before any antibacterial assay. Trypticase Soy Agar (TSA) was used for the activation of studied microorganisms, whereas Trypticase Soy Broth (TSB) was taken on for MIC and MBC determinations. Bacteria inoculum was initially prepared at McFarland 0.5, equivalent to 1.5 × 10^8^ CFU/mL.

#### 4.5.2. INT Colorimetric Assay for MIC and MBC Determinations

Bacterial susceptibility testing was performed by broth microdilution in 96-well microplates, using *para*-iodonitrotetrazolium chloride (INT) as a bacterial growth indicator. INT acts as an electron acceptor and reacts with dehydrogenases released by bacteria during the kinetic growth to form an insoluble pink-colored formazan. The assay was carried out according to the previously described protocol [29,30,31,32]. Briefly, the extracts were dissolved beforehand in the DMSO/TSB mixture. The final concentration of DMSO was less than 2.5%; this concentration is innocuous to bacterial growth. One hundred microliters of the solution were then added to the same volume of TSB in the wells of 96-well microplates followed by a two-fold serial dilution. The next step consisted of adding 100 μL of bacterial suspension (1.5 × 10^6^ CFU/mL) prepared in TSB. The plates were covered with a sterile plate sealer, then mixed by shaking for 10 min, and finally incubated at 37 °C for 18 h. The experiment was done in triplicate and repeated thrice. MIC was considered as the lowest concentration of the plant extract that prevented the visible growth of the tested bacteria. The growth of bacterial cells in each of the wells was confirmed by color change after the addition of 40 µL of INT 0.02% (w/v). In the absence of bacterial growth inhibition, the INT changed from clear to pink. Wells with DMSO alone as well as wells without any treatment were used as negative controls. Streptomycin was used as a positive control.

The MBC was determined after subculture of 50 µL (of wells content corresponding to values ≥ MIC) in 150 µL of TSB contained in new 96-well microplates, followed by incubation (37 °C) for 48 h. Then, INT was used as the abovementioned to reveal bacterial growth. The MBC was considered as the lowest concentration of the sample, which prevents a color change after the addition of INT, corresponding to the total killing effect of bacteria cells [29,30,31,32].

### 4.6. In Vitro Antiproliferative Assays

Antiproliferative properties of *E. lanceolatus* extracts were performed on four human tumor cell lines provided by the China Center for Type Culture Collection (CCTCC). These included HepG2 (human liver cancer cell line), HeLa (cervical cancer cells), HT-29 (human colon cancer cell line), and A549 (adenocarcinomic human alveolar basal epithelial cells). Cells were maintained in an adequate medium: Dulbecco’s Modified Eagle Medium (DMEM) for HepG2, Minimum Essential Medium Eagle (MEM) for HeLa, McCoy’s 5A for HT-29, and Ham’s F12 K for A549. Each culture medium was supplemented with 10% FBS, glutamine (2 mM) and 1% penicillin (100 U/mL)-streptomycin (100 µg/mL). Moreover, the cell lines were sub-cultured twice a week and incubated in a moistened atmosphere at 37 °C with 5% CO_2_ and 90% relative humidity. Hematocytometer and phase-contrast microscopy were used for the counting of viable cells. The cells at the exponential growth phase (beyond 80% confluence) were exploited for cell antiproliferative testing [57].

SRB colorimetric assay was used to investigate the antiproliferative properties of the plant extracts on the aforementioned cell lines, according to the previously described protocol [57,58]. IC_50_ was determined and considered as the concentration of plant extract required to inhibit 50% of the cell proliferation and was calculated by plotting the percentage survival versus the concentrations, using GraphPad Prism 8.0.1. Each test sample solution was performed in triplicate, in a single experiment.

### 4.7. Statistical Analysis

All tests were done in triplicate and the data obtained from antiproliferative assays were analyzed using GraphPad Prism 8.0.1 software (GraphPad., San Diego, CA, USA).

## 5. Conclusions

Our findings revealed the antibacterial potential and antiproliferative activities of *E. lanceolatus* crude methanolic extract and subsequent partition fractions (*n*-hexane, dichloromethane, ethyl acetate, and *n*-butanol). The ethyl acetate fraction was found to be the most effective one, and 32 phytochemicals have been identified in this fraction, of which quinic acid and derivatives, dihydrokaempferol, naringenin-7-*O*-glucoside, apigenin-7-*O*-glucoside, naringin, apigenin, rhoifolin, coniferyl aldehyde, and secoisolariciresinol are well documented for their antimicrobial and antiproliferative properties. These make substantial evidence to consider *E. lanceolatus* as a plant of biological importance, a valuable source for anti-infective and antitumor agents.

## Figures and Tables

**Table 1:** MIC and MBC (µg/mL) of *E. lanceolatus* extracts.

Bacteria Strains		MIC and MBC (µg/mL) of *Echinops lanceolatus* Extracts *					ATB
		MeOH	Hex	DCM	EA	BuOH	STR
Gram-negative	*Salmonella enterica*	1024	512	512	256	1024	4
	*Escherichia coli*	>1024	>1024	1024	1024	512	8
	*Enterobacter cloacae*	1024	1024	1024	512	1024	64
	*Klebsiella pneumoniae*	1024	1024	1024	>1024	>1024	>256
	*Pseudomonas aeruginosa*	>1024	>1024	>1024	1024	>1024	>256
	*Acinetobacter baumannii*	>1024	>1024	>1024	1024	>1024	256
Gram-positive	*Staphylococcus aureus*	1024	1024	512	512 (1024) ^B^	1024	4
	*Enterococcus faecalis*	>1024	>1024	>1024	>1024	>1024	256

* Each fraction tested in triplicate, at 1024 µg/mL. Reference antibiotic (ATB) Streptomycin (STR) tested at 256 µg/mL. MIC: Minimal Inhibitory Concentration; MBC: Minimal Bactericidal Concentration. ^B^: Bactericidal effect (MBC/MIC < 4). MeOH: Methanol, Hex: *n*-Hexane, DCM: Dichloromethane, EA: Ethyl acetate, BuOH: *n*-Butanol.

**Table 2:** Identified compounds from the *E. lanceolatus* EA fraction corresponding to the base peak chromatogram (BPC) in Figure 3.

Peak	Rt (min)	[M-H]^−^ (*m/z*)	MF	MS^2^ (*m/z*)	Identification *	Ref
1	1.16	117.0180	C_4_H_6_O_4_	117.0179, 99.0074, 73.0282	Succinic acid	[9]
2	1.22	191.0551	C_7_H_12_O_6_	191.0552, 173.0453, 127.0388, 93.0333, 85.0282	Quinic acid	[10]
3	1.34	153.0181	C_7_H_6_O_4_	152.9947, 109.0282, 108.0201	Dihydroxybenzoic acid	[11]
4	1.53	167.0338	C_8_H_8_O_4_	167.0334, 152.0104, 123.0440, 108.0204, 91.0177	Hydroxy-methoxy-benzoic acid isomer 1	[11]
5	1.69	179.0339	C_9_H_8_O_4_	179.0553, 135.0442	Dihydroxycinnamic acid isomer 1	[11]
6	2.15	167.0338	C_8_H_8_O_4_	167.0334, 152.0104, 123.0440, 108.0204, 91.0177	Hydroxy-methoxy-benzoic acid isomer 2	[11]
7	2.44	179.0338	C_9_H_8_O_4_	179.0553, 135.0442	Dihydroxycinnamic acid isomer 2	[11]
8	3.08	163.0389	C_9_H_8_O_3_	163.0390, 120.0523, 119.0490	Coumaric acid isomer 1	[11]
9	3.29	163.0389	C_9_H_8_O_3_	163.0390, 120.0523, 119.0490	Coumaric acid isomer 2	[11]
10	4.58	187.0965	C_9_H_16_O_4_	187.0963, 125.0960, 97.0643	Nonanedioic acid	[12]
11	5.70	137.0231	C_7_H_6_O_3_	137.0232, 93.0333, 71.4599	3-Hydroxybenzoic acid	[11]
12	5.97	337.0924	C_16_H_18_O_8_	191.0552, 173.0444, 163.0389, 119.0489, 93.0332	5-Coumaroylquinic acid	[13]
13	6.67	367.1027	C_17_H_20_O_9_	367.1055, 193.0497, 191.0553, 173.0444, 134.0361, 93.0330	5-Feruloyl quinic acid	[14]
14	7.67	515.1187	C_25_H_24_O_12_	353.0876, 191.0553, 179.0340, 161.0234, 135.0439, 93.0332, 85.0282	Dicaffeoylquinicacid isomer 1	[15]
15	7.94	515.1186	C_25_H_24_O_12_	353.0874, 191.0552, 179.0340, 173.0445, 135.0439, 93.0332, 85.0282	Dicaffeoylquinicacid isomer 2	[15]
16	8.29	515.1189	C_25_H_24_O_12_	515.1190, 353.0875, 191.0552, 179.0340, 173.0445, 161.0233, 135.0439	Dicaffeoylquinicacid isomer 3	[15]
17	8.72	515.1189	C_25_H_24_O_12_	515.1190, 353.0875, 191.0552, 179.0340, 173.0445, 135.0439, 93.0333	Dicaffeoylquinicacid isomer 4	[15]
18	9.04	287.0556	C_15_H_12_O_6_	287.0921, 259.0608, 243.0658, 201.0549, 177.0547, 151.0025, 125.0231	Dihydrokaempferol	[16]
19	9.33	433.1134	C_21_H_22_O_10_	433.1120, 313.0716, 271.0608, 177.0181, 151.0025, 119.0489, 107.0125	Naringenin-7-*O*-glucoside	[17]
20	9.59	431.0977	C_21_H_20_O_10_	431.0980, 269.0441, 268.0373, 240.0422, 211.0392, 151.0025, 117.0333	Apigenin-7-*O*-glucoside	[18]
21	10.53	177.0546	C_10_H_10_O_3_	177.0547, 162.0312, 145.0283, 133.0283, 123.0439, 121.0282, 117.0333	Coniferyl aldehyde	[19]
22	10.61	579.1503	C_27_H_32_O_14_	579.1474, 307.0825, 271.0610, 145.0283, 119.0490, 117.0334	Naringin	[20]
23	10.86	577.1346	C_27_H_30_O_14_	577.1351, 431.0982, 269.0453, 145.0284, 117.0333	Rhoifolin	[20]
24	11.02	269.0451	C_15_H_10_O_5_	269.0451, 225.0551, 151.0025, 117.0332	Apigenin	[21]
25	11.12	329.2328	C_18_H_34_O_5_	329.2331, 311.2234, 229.1438, 211.1332, 171.1016, 139.1117, 99.0802	(-)-Pinellic acid	/
26	11.87	361.0818	C_20_H_26_O_6_	361.0820, 288.0636, 269.0451, 163.0390, 145.0283, 117.0333	(-)-Secoisolariciresinol	/
27	13.09	293.2117	C_18_H_30_O_3_	293.2119, 265.2168	13-Keto-9Z,11E-octadecadienoic acid	/
28	13.45	295.2273	C_18_H_32_O_3_	295.2273, 251.2378	13S-Hydroxyoctadecadienoic acid	/
29	13.69	275.1647	C_17_H_24_O_3_	275.1648, 215.1434, 59.0125	Cyclandelate	/
30	14.17	277.2166	C_18_H_30_O_2_	277.2166	α-Linolenic acid	/
31	14.63	255.2324	C_16_H_32_O_2_	255.2326	Ethyl myristate	/
32	14.74	281.2479	C_18_H_34_O_2_	281.1389	Oleic acid	/

MF: Molecular Formula. Rt: Retention time. Ref: Reference. * Compounds corresponding to Peaks 1 to 24 were identified by comparison of the mass spectra with the available standards from databases (ChemSpider, mzCloud, mzVault, HDMB, and PubChem) and the literature data, whilst identification of Peaks 25 to 32 was only based on available standards from databases.

**Table 3:** Gradient condition for UPLC–ESI–MS/MS analysis.

Time	Phase A (5mM Ammonium Acetate, pH 9)	Phase B (50% ACN, 10mM Ammonium Acetate, pH 9)
0.00	98%	2%
1.50	98%	2%
12.00	0%	100%
14.00	0%	100%
14.10	98%	2%
16.00	98%	2%

## References

[1] Bray F., Ferlay J., Soerjomataram I., Siegel R.L., Torre L.A., Jemal A. (2018). Global cancer statistics 2018: GLOBOCAN estimates of incidence and mortality worldwide for 36 cancers in 185 countries. CA Cancer J. Clin..

[2] World Health Organization (2017). Prioritization of Pathogens to Guide Discovery, Research and Development of New Antibiotics for Drug-Resistant Bacterial Infections, Including Tuberculosis.

[3] Yu Z., Tang J., Khare T., Kumar V. (2020). The alarming antimicrobial resistance in ESKAPEE pathogens: Can essential oils come to the rescue?. Fitoterapia.

[4] Chen G., Huang B.X., Guo M. (2018). Current advances in screening for bioactive components from medicinal plants by affinity ultrafiltration mass spectrometry. Phytochem. Anal..

[5] Sarker S.D., Nahar L. (2018). Computational Phytochemistry.

[6] Bitew H., Hymete A. (2019). The genus *Echinops*: Phytochemistry and biological activities: A review. Front. Pharm..

[7] Royal Botanical Garden (KEW) *Echinops mildbraedii* (*Echinops lanceolatus*). http://www.plantsoftheworldonline.org/taxon/urn:lsid:ipni.org:names:202586–1.

[8] Roskov Y., Ower G., Orrell T., Nicolson D., Bailly N., Kirk P.M., Bourgoin T., DeWalt R.E., Decock W., Nieukerken E. (2019). Species 2000 & ITIS Catalogue of Life, 2019 Annual Checklist.

[9] Liu W.P., Li C.Y., Huang J., Liao J.Z., Ma W.J., Chen H.Y., Rui W. (2017). Identification of biomarkers in urine of rats with spleen Qi deficiency and biological significance. Zhongguo Zhong Yao Za Zhi Zhongguo Zhongyao Zazhi China J. Chin. Mater. Med..

[10] Deshpande S., Matei M.F., Jaiswal R., Bassil B.S., Kortz U., Kuhnert N. (2016). Synthesis, structure, and tandem mass spectrometric characterization of the diastereomers of quinic acid. J. Agric. Food Chem..

[11] Gruz J., Novák O., Strnad M. (2008). Rapid analysis of phenolic acids in beverages by UPLC–MS/MS. Food Chem..

[12] Bondia-Pons I., Barri T., Hanhineva K., Juntunen K., Dragsted L.O., Mykkänen H., Poutanen K. (2013). UPLC-QTOF/MS metabolic profiling unveils urinary changes in humans after a whole grain rye versus refined wheat bread intervention. Mol. Nutr. Food Res..

[13] Clifford M.N., Johnston K.L., Knight S., Kuhner N. (2003). Hierarchical scheme for LC-MSn identification of chlorogenic acids. J. Agric. Food Chem..

[14] Kuhnert N., Jaiswal R., Matei M.F., Sovdat T., Deshpande S. (2010). How to distinguish between feruloyl quinic acids and isoferuloyl quinic acids by liquid chromatography/tandem mass spectrometry. Rapid Commun. Mass Spectrom..

[15] Zhong R.F., Xu G.B., Wang Z., Wang A.M., Guan H.Y., Li J., He X., Liu J.H., Zhou M., Li Y.J. (2015). Identification of anti-inflammatory constituents from *Kalimeris indica* with UHPLC-ESI-Q-TOF-MS/MS and GC–MS. J. Ethnopharmacol..

[16] Moqbel H., El Hawary S.S.E.D., Sokkar N.M., El-Naggar E.M.B., El Boghdady N., El Halawany A.M. (2018). HPLC-ESI-MS/MS characterization of phenolics in *Prunus amygdalus*, cultivar “umm alfahm” and its antioxidant and hepatoprotective activity. J. Food Meas. Charact..

[17] Kammerer B., Kahlich R., Biegert C., Gleiter C.H., Heide L. (2005). HPLC-MS/MS analysis of willow bark extracts contained in pharmaceutical preparations. Phytochem. Anal..

[18] Song H.P., Zhang H., Fu Y., Mo H.Y., Zhang M., Chen J., Li P. (2014). Screening for selective inhibitors of xanthine oxidase from *Flos chrysanthemum* using ultrafiltration LC–MS combined with enzyme channel blocking. J. Chromatogr. B.

[19] Mena P., Sánchez-Salcedo E.M., Tassotti M., Martínez J.J., Hernández F., Del Rio D. (2016). Phytochemical evaluation of eight white (*Morus alba* L.) and black (*Morus nigra* L.) mulberry clones grown in Spain based on UHPLC-ESI-MS^n^ metabolomic profiles. Food Res. Int..

[20] Barreca D., Bellocco E., Caristi C., Leuzzi U., Gattuso G. (2010). Flavonoid composition and antioxidant activity of juices from chinotto (*Citrus myrtifolia* Raf.) fruits at different ripening stages. J. Agric. Food Chem..

[21] Arivalagan M., Roy T.K., Yasmeen A.M., Pavithra K.C., Jwala P.N., Shivasankara K.S., Manikantan M.R., Hebbar K.B., Kanade S.R. (2018). Extraction of phenolic compounds with antioxidant potential from coconut (*Cocos nucifera* L.) testa and identification of phenolic acids and flavonoids using UPLC coupled with TQD-MS/MS. LWT.

[22] Seukep A.J., Kuete V., Nahar L., Sarker S.D., Guo M. (2019). Plant-derived secondary metabolites as the main source of efflux pump inhibitors and methods for identification. J. Pharm. Anal..

[23] Kuete V. (2010). Potential of Cameroonian plants and derived products against microbial infections: A review. Planta Med..

[24] Liu Y., Cao Y., Wang T., Dong Q., Li J., Niu C. (2019). Detection of 12 common food-borne bacterial pathogens by TaqMan Real-Time PCR using a single set of reaction conditions. Front. Microbiol..

[25] Nikaido H. (1994). Prevention of drug access to bacterial targets: Permeability barriers and active efflux. Science.

[26] Wright G.D. (2005). Bacterial resistance to antibiotics: Enzymatic degradation and modification. Adv. Drug Deliv. Rev..

[27] Kuete V., Efferth T. (2015). African flora has the potential to fight multidrug resistance of cancer. Biomed Res. Int..

[28] Bruneton J. (1999). Pharmacognosie: Phytochimie, Plantes Médicinales.

[29] Seukep A.J., Fankam A.G., Djeussi D.E., Voukeng I.K., Tankeo S.B., Noumedem J.A.K., Kuete A.H., Kuete V. (2013). Antibacterial activities of the methanol extracts of seven Cameroonian dietary plants against bacteria expressing MDR phenotypes. Springerplus.

[30] Seukep A.J., Ngadjui B.T., Kuete V. (2015). Antibacterial activities of *Fagara macrophylla*, *Canarium schweinfurthii*, *Myrianthus arboreus*, *Dischistocalyx grandifolius* and *Tragia benthamii* against multi-drug resistant Gram-negative bacteria. Springerplus.

[31] Seukep A.J., Sandjo L.P., Ngadjui B.T., Kuete V. (2016). Antibacterial and antibiotic-resistance modifying activity of the extracts and compounds from *Nauclea pobeguinii* against Gram-negative multi-drug resistant phenotypes. BMC Complement. Altern. Med..

[32] Seukep A.J., Sandjo L.P., Ngadjui B.T., Kuete V. (2016). Antibacterial activities of the methanol extracts and compounds from *Uapaca togoensis* against Gram-negative multi-drug resistant phenotypes. S. Afr. J. Bot..

[33] Vandal J., Abou-Zaid M.M., Ferroni G., Leduc L.G. (2015). Antimicrobial activity of natural products from the flora of Northern Ontario, Canada. Pharm. Biol..

[34] Kima J.E., Seob J.H., Baec M.S., Baed C.-S., Yooe J.C., Bangf M.A., Choa S.S., Park D.H. (2016). Antimicrobial constituents from *Allium hookeri* Root. Nat. Prod. Commun..

[35] Purohit A., Mohan A. (2019). Antimicrobial effects of pyruvic and succinic acids on *Salmonella* survival in ground chicken. LWT.

[36] Kumar R., Chandar B., Parani M. (2018). Use of succinic and oxalic acid in reducing the dosage of colistin against New Delhi metallo-β-lactamase- bacteria. Indian J. Med. Res..

[37] Gaglione M., Malgieri G., Pacifico S., Severino V., D’Abrosca B., Russo L., Fiorentino A., Messere A. (2013). Synthesis and biological properties of caffeic acid-PNA dimers containing guanine. Molecules.

[38] Pei K., Ou J., Huang J., Ou S. (2016). *p*-Coumaric acid and its conjugates: Dietary sources, pharmacokinetic properties and biological activities. J. Sci. Food Agric..

[39] Abdallah H.M., Ezzat S.M., Dine R.S., Abdel-Sattar E., Abdel-Naim A.B. (2013). Protective effect of *Echinops galalensis* against CCl4-induced injury on the human hepatoma cell line (Huh7). Phytochem. Lett..

[40] Fraisse D., Felgines C., Texier O., Lamaison J.L. (2011). Caffeoyl derivatives: Major antioxidant compounds of some wild herbs of the *Asteraceae* family. Food Nutr. Sci..

[41] Mijangos-Ramos I.F., Zapata-Estrellaa H.E., Ruiz-Vargas J.A., Escalante-Erosa F., Gómez-Ojeda N., García-Sosa K., Cechinel-Filho V., Meira-Quintão N.L., Pena-Rodríguez L.M. (2018). Bioactive dicaffeoylquinic acid derivatives from the root extract of *Calea urticifolia*. Rev. Bras. Farm..

[42] Petropoulos S.A., Pereira C., Tzortzakis N., Barros L., Ferreira I.C.F.R. (2018). Nutritional value and bioactive compounds characterization of plant parts from *Cynara cardunculus* L. (*Asteraceae*) cultivated in Central Greece. Front. Plant Sci..

[43] Zhang Y., Yan G., Sun C., Li H., Fu Y., Xu W. (2018). Apoptosis effects of dihydrokaempferol isolated from *Bauhinia championii* on Synoviocytes. Evid. Based Complement. Altern. Med..

[44] Harborne J.B. (2017). The Flavonoids Advances in Research Since 1986.

[45] Wang Y., Xu Z., Huang Y., Wen X., Wu Y., Zhao Y., Ni Y. (2018). Extraction, purification, and hydrolysis behavior of Apigenin-7-*O*-glucoside from *Chrysanthemum morifolium* tea. Molecules.

[46] Wang H., Li Y.L., She W.Z., Guo G.Q., Jiang Z.Y., Cen Y.Z., Fan Z.Y. (2007). Studies on antiproliferative effect of flavones compounds isolated from Yao herb medicines. Zhong Yao Cai.

[47] Smiljkovic M., Stanisavljevic D., Stojkovic D., Petrovic I., Vicentic J.M., Popovic J., Grdadolnik S.G., Markovic D., Sankovic-Babice S., Glamoclija J. (2017). Apigenin-7-*O*-glucoside versus apigenin: Insight into the modes of anticandidal and cytotoxic actions. EXCLI J..

[48] Chen R., Qi Q.L., Wang M.T., Li Q.Y. (2016). Therapeutic potential of naringin: An overview. Pharm. Biol..

[49] Yan X., Qi M., Li P., Zhan Y., Shao H. (2017). Apigenin in cancer therapy: Anti-cancer effects and mechanisms of action. Cell Biosci..

[50] Salehi B., Venditti A., Sharifi-Rad M., Kregiel D., Sharifi-Rad J., Durazzo A., Lucarini M., Santini A., Souto E.B., Novellino E. (2019). The therapeutic potential of apigenin. Int. J. Mol. Sci..

[51] Refaat J., Desoukey Y.S., Ramadan M.A., Kamel M.S. (2015). Rhoifolin: A review of sources and biological activities. IJP.

[52] Xu J.P. (2016). Cancer Inhibitors from Chinese Natural Medicines.

[53] Céspedes C.L., Avila J.G., García A.M., Becerra J., Flores C., Aqueveque P., Bittner M., Hoeneisen M., Martinez M., Silva M. (2006). Antifungal and antibacterial activities of *Araucaria araucana* (Mol.) K. Koch heartwood lignans. Zeitschrift Naturforschung C.

[54] Kezimana P., Dmitriev A.A., Kudryavtseva A.V., Romanova E.V., Melnikova N.V. (2018). Secoisolariciresinol diglucoside of flaxseed and its metabolites: Biosynthesis and potential for nutraceuticals. Front. Genet..

[55] Dilika F., Bremner P.D., Meyer J.J. (2000). Antibacterial activity of linoleic and oleic acids isolated from *Helichrysum pedunculatum*: A plant used during circumcision rites. Fitoterapia.

[56] Parsaee H., Asilib J., Mousavic S.H., Soofi H., Emami S.A., Tayarani-Najarane Z. (2013). Apoptosis induction of *Salvia chorassanica* root extract on human cervical cancer cell line. Iran J. Pharm. Res..

[57] Chen G., Li X., Saleri F., Guo M. (2016). Analysis of flavonoids in *Rhamnus davurica* and its antiproliferative activities. Molecules.

[58] Dzoyem J.P., Guru S.K., Pieme C.A., Kuete V., Sharma A., Khan I.A., Saxena A.K., Vishwakarma R.A. (2013). Cytotoxic and antimicrobial activity of selected Cameroonian edible plants. BMC Complement. Altern. Med..

